# A Practical Method to Assess Bird Strike Risk in Air Operations Using a Count‐Based Risk Mitigation Tool

**DOI:** 10.1111/risa.70101

**Published:** 2025-09-02

**Authors:** A. Marijn Teunizen, Hans van Gasteren, Karen L. Krijgsveld

**Affiliations:** ^1^ Royal Netherlands Air Force Breda the Netherlands; ^2^ Theoretical and Computational Ecology Institute for Biodiversity and Ecosystem Dynamics, University of Amsterdam Amsterdam the Netherlands; ^3^ Wageningen Environmental Research Wageningen University & Research Wageningen the Netherlands

**Keywords:** aviation, bird control, bird counts, bird strike risk, bird strike, wildlife hazard management

## Abstract

Bird strikes pose a risk to aviation. Collisions between birds and airplanes result in a threat to human lives, economic losses, and material damage. The majority of these collisions occur on airfields during takeoff and landing. Knowing what bird species are present on airfields, in what numbers, and relating that to the extent to which these birds are involved in collisions can help to direct bird control activities to specific bird species and thus reduce bird strikes. In this article, we offer a method to quantify the risk of bird strikes at airfields based on counts of bird abundance on airfields. We analyzed bird abundance in relation to bird strike risks based on a dataset from six Dutch airfields covering three decades. We used the data to define two metrics: Species Strike Impact (SSI) and Bird Strike Risk Index (BSRI), which are both independent of aspects such as bird behavior, habitat, season, or weather. These two metrics, respectively, reflect the bird strike risk per individual of a bird species on an airfield based on hazard probability and severity (SSI), and they provide quick insight in the local status of overall bird strike risks by summing all species‐related risks into one overall index (BSRI). Both metrics are calculated from counts on the airfield of birds, bird strikes, and air traffic movements. This method can be readily incorporated as a leading indicator in flight safety management at airfields, enabling bird control personnel to take risk‐reducing actions targeted at specific bird species on airfields.

## 1 Introduction

Airfields worldwide consist largely of grassland and thus provide a niche for all kinds of wildlife, ranging from insects to birds and mammals (Blackwell et al. 2013; Conkling et al. 2018). Military airbases specifically provide habitats for a range of species given that these airbases are situated on less productive grounds often combined with security measures that require forested boundaries surrounding grasslands of the airfield (van Heusden et al. 2023). Wildlife, in particular birds, poses a considerable safety and financial burden to aviation (Allan 2002; El‐Sayed 2019). Every year, collisions between birds and airplanes occur. The majority of these occur at low altitudes, during takeoff and landing on airfields (Metz et al. 2020). Collisions can result in a threat to human lives, economic losses, and material damage (Richardson and West 2000; Allan 2002; Anderson et al. 2015; Thorpe 2016; van Gasteren et al. 2019). Nevertheless, the ecological characteristics (e.g., grassland surrounded by forest) of an operational airfield create a suitable habitat. Therefore, any airfield will be faced with the presence of birds (Dekker and Buurma 2003). Controlling the risk of bird strikes in aviation is thus an important aspect of flight safety (ICAO 2020).

The analysis of risk has become increasingly important over many years and requires adequate and clear formulation of the methodology (Kaplan and Garrick 1981; Haimes 2009; Andretta 2014; Aven and Zio 2014). In flight safety, a hazard is commonly defined as a situation or condition with the potential to cause adverse effects (Kaplan and Garrick 1981; ICAO 2020). In the context of bird strike management, a hazard can be referred to as bird presence (ICAO 2020). Subsequently, a risk of a hazard is defined as a measure of likelihood of a hazard occurring and is dependent on both the probability and the severity of adverse effects (Kaplan and Garrick 1981; Aven and Zio 2014; ICAO 2020). Risk analysis is based on the following three questions: What can happen (a hazard)? How likely is it that that will happen (hazard probability)? And if it does happen, what are the consequences (hazard severity)? (Kaplan and Garrick 1981; Aven and Zio 2014). To find answers to these questions in the process of risk analysis, two variables are essential: (1) the hazard probability, or the likelihood that a hazard will occur, and (2) hazard severity, or the extent of harm expected as a consequence of the identified hazard. In other words, birds that are abundant on an airfield pose a hazard risk but not all bird species have the same risk in terms of flight safety due to variation in hazard probability and severity. For example, a strike with an individual small passerine does not result in damage and is not considered a major hazard, and thus, passerines are considered to be low risk, whereas a strike with a goose can cause considerable damage and is considered a major risk in terms of flight safety. The hazard probability is dependent on air traffic movements, the probability of a bird species to collide with an aircraft, and the number and species of birds present at the airfield (Dolbeer et al. 2000; Allan et al. 2003; Zakrajsek and Bissonette 2005; Allan 2006; Soldatini et al. 2010). In addition, to understand species composition and abundance on an airfield, knowledge is required of habitat preferences in relation to landscape and environmental conditions (Soldatini et al. 2010; Blackwell et al. 2013). Hazard severity is dependent on mass of the bird and the size of bird groups. As measure of hazard severity, body weight of a bird species can be used, as body weight correlates strongly with impact (Dolbeer et al. 2000; DeVault et al. 2011, 2018). To control and mitigate the risk in terms of bird strike management, airfields generally use safety performance indicators. Of these, leading (or proactive) indicators look ahead and attempt to assess risks and therewith prevent hazards from occurring. Lagging (or reactive) indicators, on the other hand, focus on hazards that occurred in the past and provide a tool to understand and analyze trends and history of these hazardous events. Leading indicators are difficult to quantify because of their predictive nature, but they form an important addition to lagging indicators. Leading indicators are useful for air operation managers and bird control units because they allow risk‐mitigating actions to be taken a priori. For example, a bird control unit can act on a leading indicator that predicts a higher risk, by increasing the number of airfield inspections and taking targeted measures to mitigate the risk. Both leading and lagging measures are essential in bird strike management. Sometimes even a species‐specific protocol for risk mitigation is required (MacKinnon 2004; Soldatini et al. 2010).

Risk analysis in bird strike management has led to a number of methods to describe and assess risk (Table 1), both as lagging and leading indicators, and based on different types of data. One of the main conclusions from these studies is that heavy animals or birds are most hazardous to aircrafts (Dolbeer et al. 2000; DeVault et al. 2011, 2018; Nilsson et al. 2021). Additionally, only a few methods provide leading indicators, and only a few were validated, where the studies of Soldatini et al. (2011) and Gutiérrez Serralde et al. (2023) are most comparable to ours. The main difference between these two studies and ours is that bird strike probability was summarized for 15 ecological bird groups in those two studies, whereas we calculate these probabilities for individual species. Andrews et al. (2022) developed a dynamic, evidence‐based risk model based on three techniques in an Australian airport, where these three distinct techniques predict the likelihood of bird strike, but for only three hazardous species. Chen et al. (2018) developed a decision‐making method that initiates bird‐repelling actions based on sensors detecting birds flying in the airport environment. Studies that focus on preventing bird strike risk during the en‐route phase of flight are beyond the scope of this study. These so‐called bird avoidance systems do not aim to reduce the number of (hazardous) birds on an airport but to restrict air operations in areas with high bird densities (e.g., Kelly 2002; van Gasteren et al. 2019).

**TABLE 1:** Overview of published bird strike risk analysis methods.

Study	Indicator	Bird abundance	Bird strikes	Relative or absolute to local situation	Validated
Dolbeer et al. (2000)	Lagging	No	Yes	Relative	No
Morgenroth (2003)	Leading	No	No	Relative	No
Allan et al. (2003)	Lagging	No	No	Relative	No
Allan et al. (2006)	Lagging	No	Yes	Relative	No
Both et al. (2010)	Leading	Yes	Yes	Relative	No
Soldatini et al. (2010)	Leading	Yes	Yes	Absolute	Yes
Soldatini et al. (2011)	Leading	Yes	Yes	Absolute	Yes
DeVault et al. (2011)	Lagging	No	Yes	Relative	No
Devault et al. (2018)	Lagging	No	Yes	Relative	Yes
Chen et al. (2018)	Leading	No	No	Absolute	No
Hu et al. (2020)	Lagging	Yes	No	Relative	No
Nilsson et al. (2021)	Leading	Yes	Yes	Relative	No
Andrews et al. (2022)	Leading	Yes	Yes	Absolute	No
Gutiérrez Serralde et al. (2023)	Leading	Yes	Yes	Absolute	Yes
*This study*	*Leading*	*Yes*	*Yes*	*Absolute*	*Yes*

*Note*: Shown for each study are whether metrics are leading or lagging; the data used (i.e., input of bird abundance and strike data); whether variables incorporated in the algorithm are relative (i.e., interpreted in relation to another value or context, e.g., ranking, percentage) to local situation) or absolute (i.e., fixed, not changing regardless of the context); and if the method is validated. For comparison, values for the method presented in this article are included in italic at the bottom of the table.

In this article, we provide a method to anticipate and mitigate bird strike risk, based on the abundance of birds at an airfield. At airbases of the Royal Netherlands Air Force (RNLAF), standardized bird counts have been carried out for nearly three decades, resulting in a large dataset on airfield bird abundance and species composition. Together with data collected on aircraft movements and bird strikes, this provides a means to relate data on bird strike probability and severity to the data on bird abundance. If birds are on the airfield (i.e., they are counted during bird counts on the airfield), then they pose a known risk to aircraft operations (known because it is related to body mass (Dolbeer et al. 2000; DeVault et al. 2011, 2018) and strike probability (Kelly et al. 2001; Soldatini et al. 2011; Blackwell et al. 2019)). Because this risk is known, we can use the bird counts to warn bird control units of increased risks. For example, if the numbers of common buzzard (*Buteo buteo*) increase above a specific level and/or for several weeks in a row, then bird control units can be (automatically) alerted of this, and they can take directed actions to reduce the number of common buzzards back to acceptable levels. Common buzzards are rather heavy birds and are often involved in bird strikes. The known hazard risk based on strike probability and severity of the common buzzard is translated into a species‐specific metric, the Species Strike Impact (SSI). This metric simply reflects the hazard risk of having a common buzzard on an airfield, because in SSI the body mass of the common buzzard is combined with its strike probability. If numbers of common buzzards increase, the strike probability increases. Similarly, besides common buzzards, multiple other species can occur at airfields in varying numbers. To quantify the overall risk of all these birds together, we combine the SSI metrics of all species occurring on the airfield with their abundance, resulting in the Bird Strike Risk Index (BSRI). This results in a general metric that indicates if any of the species is above acceptable levels. For example, common buzzards have a relatively high strike probability and severity. Thus, low numbers contribute to a high BSRI. On the other hand, Western Jackdaws (*Coloeus monedula*) have a relatively low strike probability and severity, and thus high numbers contribute to a lower BSRI. This way, we use bird abundance to quantify hazard risk and prevent hazards from occurring and therewith offer a method for bird strike management that is based on leading indicators and that is applicable to any airfield at any time.

Here, we first present our mathematical approach to quantify Species Strike Impact (SSI) and BSRI. In addition, we present a validation of both indices against bird status issued by local bird control units and used inspection counts to calculate standardized local bird status. With this method, we provide a bird‐count‐based risk mitigation tool that forms an effective warning system for potential bird strikes by combining both probability and severity of these bird strikes.

## 2 Materials and Methods

Bird count data were systematically recorded by bird control units on six military airbases in the Netherlands, which were all used by multiple types of aircraft. Two airbases are primarily used for relatively slow‐moving helicopter operations (De Kooy, Gilze‐Rijen), two for fighter jet operations (Leeuwarden, Volkel), one for training aircraft (Woensdrecht), and one for aircraft transport and air carrier operations as well as civil operations (Eindhoven). Because strike probability and severity are related strongly to bird species (and less to aircraft type, see Section 4 and Pfeiffer et al. 2018), and because we aim to address the aspect of risk related to birds, all six airbases were grouped in one dataset. Bird strike probability is also related to aircraft, but to a much lesser extent than bird species. Strikes with non‐bird species were very limited (1.8%) and were therefore excluded from our analyses.

### 2.1 Bird Count Data

Bird abundance is vital information for bird strike prevention measures. Therefore, bird control units of the RNLAF systematically counted numbers of birds per species on the entire airfield. Counts were carried out by car, following a fixed transect, and are done year‐round at least two times a week. Bird numbers were summed per species in predefined plots on the airbase. Only birds that use the airbase were counted, including, for example, hovering kestrels, but not geese flocks flying overhead. Data on bird counts were collected over the period 1995–2022 (De Kooy since 2005) and stored in a database. To express bird abundance, the mean number per species per 10 ha was calculated.

Besides systematic counts of birds on the airfield, a second dataset of bird data on the same airbases was available to use for validation. These are data from runway inspection bird counts. Runway inspections were carried out by bird control units 4–6 times a day to check for birds in the runway area, by driving down all the runways and taxi tracks. Any actions required to expel birds were carried out during these inspections. Birds in the runway area were counted in number classes per species (1–2, 3–5, 6–10, 11–25, 26–50, 51–100, 101–250, 251–500, 501–1000, and >1000). Inspection count data were collected over the period 2000–2022 (De Kooy since 2005) and stored in a database. At each inspection, bird control personnel assessed the local bird status as being normal, alert, or critical, based on their experience of bird species and numbers in the runway area, and aircraft movements. Inspection counts with unknown local bird status were removed from the dataset (nocturnal and low visibility inspections).

### 2.2 Bird Strike Data

The data on bird strikes included all reported local bird strikes for the airbases of the RNLAF from 1995 to 2022 (De Kooy since 2005). The database included confirmed strikes by pilots, dead birds found on the runway, and bird strikes reported by maintenance personnel. Local bird strikes included strikes during the flight phases of final approach, landing, touch‐and‐go, takeoff, and climb. Final approach and climb phases partly match the airbases fence and represented 8.3% and 1.0% of the total number of bird strikes, respectively (Table 2). Only strikes for which the bird species involved was known were included. Bird remains were identified up to species level by bird control units or were identified through macroscopic or DNA identification by Naturalis laboratories (Naturalis Biodiversity Centre, Leiden, the Netherlands). Bird strikes with multiple numbers were treated as one bird strike. The database contained 1663 bird strikes of which 1507 were known up to species level (91%) (Table 2).

**TABLE 2:** An overview of the airbases, their size, survey periods, number of bird strikes and species level known (%), the number of bird counts and inspection counts.

Airbase	Area (ha)	Survey period	Bird strikes	Species level known (%)	Bird counts	Inspection counts
De Kooy	118.6	2005–2022	57	64.9	1714	22,637
Leeuwarden	424.2	1995–2022	285	91.2	4528	28,088
Volkel	486.5	1995–2022	221	85.5	3577	31,366
Eindhoven	526.9	1995–2022	957	94.4	4153	52,985
Gilze‐Rijen	670.7	1995–2022	65	81.5	3402	21,653
Woensdrecht	220.3	1995–2022	78	83.3	3981	24,758
**Total**	2447.7		1663	90.6	21,355	181,487

### 2.3 Aircraft Movements

Every takeoff or landing was registered as an aircraft movement. Circuits and touch‐and‐gos, which are typical training missions in military aviation, were counted for both a landing and takeoff. Military aircraft movements were evenly distributed over the year. The mean number of aircraft movement per year as well as the distribution of aircraft types is presented in Table 3.

**TABLE 3:** An overview of the airbases, mean number of aircraft movements per year, percentage distribution of aircraft movements per class (fighters, transport, helicopters, civil aviation, and unknown).

Airbase	Mean (#/year)	Fighters (%)	Transport and airline (%)	Helicopters (%)	Civil aviation and unknown (%)
De Kooy	31,064	0.2	1.4	86.6	11.8
Leeuwarden	12,403	82.4	5.4	9.9	2.3
Volkel	11,301	80.5	2.9	14.5	2.1
Eindhoven	33,717	1.6	75.2	1.7	21.6
Gilze‐Rijen	17,619	1.6	2.2	86.8	9.4
Woensdrecht	10,680	3.7	5.6	4.0	86.7
**Total**	116,785	19.5	26.2	34.3	20.0

### 2.4 Species Strike Impact

Not all species are equally sensitive to bird strikes or cause damage to the same extent. To account for these species‐specific risks, we defined the Species Strike Impact (SSI) to reflect not only the sensitivity of a bird species to collide with an aircraft but also its impact, as well as its abundance on an airfield. SSI is based on the bird strike ratio (bird strike per 10,000 aircraft movements) divided by the bird density (birds per 10 ha) of a species on the airbase, as an indicator of the relative likelihood of a particular bird species to collide with an aircraft. This number was then multiplied by the bird's body mass as indicator of damage (strike impact; for relation between body mass and strike impact, see Dolbeer et al. 2000; Allan 2006; Soldatini et al. 2010; DeVault et al. 2011; DeVault et al. 2016; Pfeiffer et al. 2018). Strike probability is also species‐specific and varies strongly between species (Kelly et al. 2001; Soldatini et al. 2011; Blackwell et al. 2019). SSI was calculated for those species for which both count data and bird strikes were available, as well as for species with a bird density ≥0.001 bird/10 ha and no reported bird strikes. In the latter case, the bird strike ratio was defined as less than one bird strike during the total aircraft movements from Table 3 and thus was set at <0.0035 bird strikes per 10,000 aircraft movements.

SSI is calculated as

$$\mathrm{SS}I_{i}=\ln\left(\frac{\mathrm{BS}\_\mathrm{rati}o_{i}}{\mathrm{bird}\_\mathrm{densit}y_{i}}+1\right)\times \mathrm{bird}\_\mathrm{bodymas}s_{i}$$

where BS_ratio*_i_* is the number of bird strikes of species *i* per 10,000 aircraft movements; bird_density*_i_* is the average number for all counts of species *i* per 10 ha and bird_bodymass*_i_* is the mean body mass of species *i* in kilogram (Dunning 2008; Storchová and Hořák 2018). Logarithm +1 was taken to normalize the count data. SSI was calculated for 97 bird species (Appendix 1).

### 2.5 Bird Strike Risk Index

We developed BSRI to identify current hazard risk levels related to birds at the airbase. In this index, the SSI metrics of all species occurring on the airfield were combined with their abundance. For BSRI, bird abundance data are combined with SSI and can be calculated from the bird counts that are available at an airfield (similar to SSI). Here, we calculated BSRI from data from runway inspection counts, because this is the easiest and most abundant type of bird count, and also because it provided a means to evaluate the method. BSRI was thus quantified as abundance per species present per runway inspection count multiplied by SSI.

BSRI is calculated as

$$\mathrm{BSRI}=\ln\left(\left(\sum_{i=1}^{i=n}n_{i}\times \mathrm{SS}I_{i}\right)+1\right)$$

where *n_i_* represents the class middle of the inspection count of species *i* and SSI*_i_* is the Species Strike Impact of species *i*. Logarithm was taken to normalize the count data. BSRI was calculated for all individual runway inspection counts in the study period.

**FIGURE 1:**
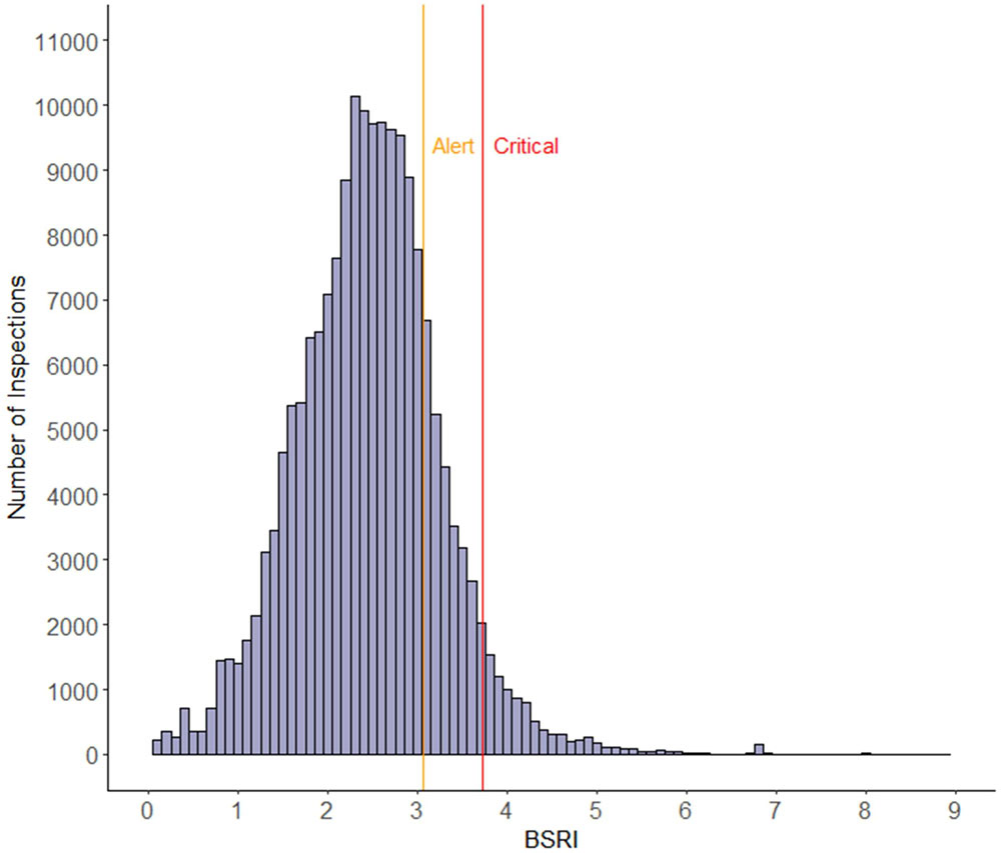
Histogram of the BSRI values of all daytime runway inspection counts on six RNLAF airbases. Orange and red lines indicate thresholds for local bird status alert (3.1 = 80% of values) and critical (3.7 = 95% of values), as calculated from BSRI values. BSRI, Bird Strike Risk Index.

A local bird status was assigned by bird control units after each runway inspection count, and these statuses could be used for validation of the BSRI. These statuses were assigned on the basis of experience (see Section 2.1), where bird numbers were perceived by experienced local bird control personnel as being reason for the airfield to go to status “alert” or “critical.” Next to this qualitative local bird status, BSRI values were used to assign a quantified local bird status, based on set thresholds. These thresholds were defined from the BSRI values of all runway inspection counts of all airbases. The top 80% threshold value of BSRI was defined as the threshold for local bird status “alert” and the top 95% threshold value as the threshold for local bird status “critical” (Figure 1). The validation was done by comparing the quantified BSRI statuses with the qualitative statuses assigned by bird control personnel, using a confusion matrix (Section 3.2 and Appendix 2).

### 2.6 Statistical Analyses

All analyses were performed using the open‐source statistical program R version 4.2.2. (R Core Team 2023). We used generalized additive models (GAMs) with a Gaussian distribution (Wood et al. 2015) with day within season to describe seasonal and yearly variation in BSRI. We used “ggplot2” package for plotting (Wickham 2016) and “mgcv” package for GAM analysis (Wood 2011).

## 3 Results

### 3.1 Species Strike Impact

SSI was calculated for 97 different bird species (Appendix 1). Canada Goose (*Branta canadensis*), White Stork (*Ciconia ciconia*), Great Black‐backed Gull (*Larus marinus*), and Rough‐legged Buzzard (*Buteo lagopus*) were species with the largest SSI, not surprising given their large body mass. European Stonechat (*Saxicola rubicola*), Fieldfare (*Turdus pilaris*), Meadow Pipit (*Anthus pratensis*), Western Yellow‐wagtail (*Motacilla flava*), and White Wagtail (*Motacilla alba*) were species with the lowest SSI values. On RNLAF airbases, common bird species that had the highest bird strike frequencies were common buzzard, common kestrel (*Falco tinnunculus*), common swift (*Apus apus*), and barn swallow (*Hirundo rustica*). Bird species that were common on the airbases but were rarely involved in strikes were Carrion Crow (*Corvus corone*), Common Starling (*Sturnus vulgaris*), Rook (*Corvus frugilegus*), and Western Jackdaw.

### 3.2 Bird Strike Risk Index

BSRI was calculated for 181,487 daytime runway inspection counts, resulting in the histogram of BSRI in Figure 1. Both 80% and 95% thresholds of all observations were calculated, respectively, representing alert and critical local bird status according to BSRI. These thresholds corresponded with a BSRI value of 3.1 and 3.7, respectively. On the basis of these thresholds, 28,169 inspections were categorized as alert and 9308 as critical. On the basis of the percentage of inspections with status critical, Leeuwarden was the most hazardous airbase (8.6% of all inspections), whereas Woensdrecht (3.5%) and Gilze‐Rijen (3.3%) were the least hazardous airbases.

The validation of local bird status as quantified with BSRI in relation to the local bird status as assigned by bird control personnel had an overall accuracy of 82.6%. Detailed statistics of the model and its performance are provided in the confusion matrix of Appendix 2.

**FIGURE 2:**
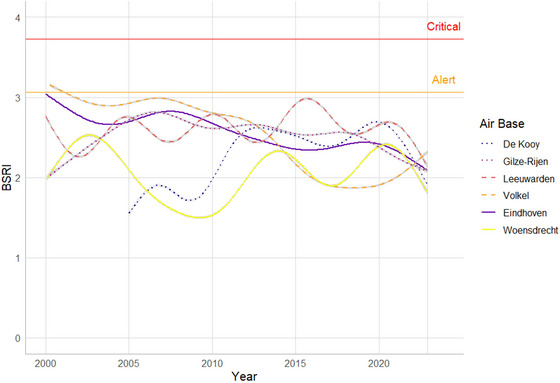
Long‐term patterns of BSRI for six RNLAF airbases from 2000 to 2022. Lines denote GAMs fitted through BSRI values per month for each airbase. BSRI, Bird Strike Risk Index.

Seen over the long term (2000–2022), the average yearly variation in BSRI per airbase fluctuated over the study period (Figure 2, Table 4). BSRI showed a significant overall increase on two airbases (De Kooy and Leeuwarden) and a decrease on three airbases (Volkel, Eindhoven, and Gilze‐Rijen). BSRI for Woensdrecht did not show a significant change. Over the last 10 years, BSRI decreased significantly on five out of six airbases (Woensdrecht non‐significant).

**TABLE 4:** Results of linear trends per year of Bird Strike Risk Index (BSRI) per airbase.

	Airbase	Trend	Adj *R* ^2^	df
Whole period—2000–2022	De Kooy	0.045^***^	0.456	213
	Leeuwarden	0.006^***^	0.049	270
	Volkel	−0.061^***^	0.824	271
	Eindhoven	−0.029^***^	0.829	274
	Gilze‐Rijen	−0.007^***^	0.045	271
	Woensdrecht	0.002	−0.003	268
Last 10 years—2013–2022	De Kooy	−0.024^***^	0.158	118
	Leeuwarden	−0.036^***^	0.293	117
	Volkel	−0.030^***^	0.172	118
	Eindhoven	−0.016^***^	0.302	118
	Gilze‐Rijen	−0.051^***^	0.816	118
	Woensdrecht	−0.003	−0.006	118

*Note*: Linear trends were determined over two periods c.q. the whole research period and the last 10 years.

*p < 0.05.

**p < 0.01.

***p < 0.001.

**FIGURE 3:**
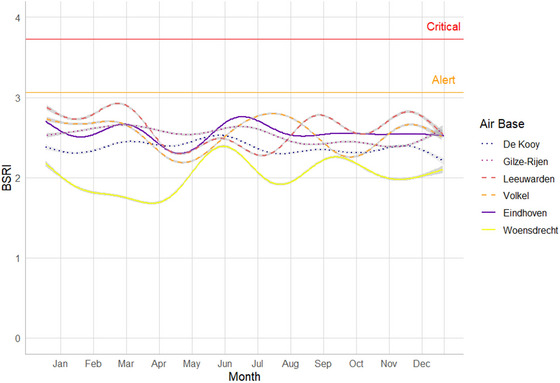
Seasonal patterns of mean BSRI for six RNLAF airbases from 2000 to 2022. Lines denote GAMs fitted through daily means. BSRI, Bird Strike Risk Index.

BSRI showed variation over the seasons and between airbases (Figure 3). BSRI values of all airbases were, however, below the thresholds alert and critical. Low values during the breeding season in May were seen in Leeuwarden, Volkel, Eindhoven, and Woensdrecht. Peaks occurred in the early or late summer months on Leeuwarden, Volkel, Eindhoven, and Woensdrecht. BSRI at helicopter fields De Kooy and Gilze‐Rijen was relatively stable throughout the year.

## 4 Discussion

During air operations, safety in relation to bird strike hazards faces the problem of a diverse bird species population with their own ecological and behavioral characteristics. In this article, we provide a method to quantitatively express bird strike risks. Our method includes data on bird abundance, bird strikes, and air traffic movements, and has no relative variables and can act as a leading indicator in bird strike management, as expressed in Table 1.

### 4.1 Use of SSI to Reflect Bird Strike Risks

In this article, we present the SSI metric to classify bird species from high to low hazard risk. We calculated SSI for European species. However, SSI can be easily calculated for other continents or countries as well, if species‐specific data on bird strikes and bird abundance are available (see, e.g., Nilsson et al. 2021). To show how the SSI risk classification can be used, we provide some examples of species‐specific bird strike probabilities. Corvidae, such as Carrion Crow, Rook, and Magpie, posed a very low risk for flight safety (based on strike data), and this was reflected in low SSI values. These species were common during runway inspections but hardly ever collided with aircraft (Dekker et al. 2003; MacKinnon 2004), because of their cognitive abilities enabling them to avoid threats while foraging at an airfield (Marzluff and Swift 2017; Lee and Thornton 2021; Wink 2022). Species that were relatively prone to bird strikes were owls and birds of prey. For owls, we generally lack bird count data (rare occurrence) rendering the bird strike sensitivity values unreliable. For birds of prey, such as common buzzard and kestrel, but certainly also the less common harriers and other species of falcon, high SSI values are based on large datasets. In combination with their considerable body mass, the common buzzard is one of the most hazardous species while also one of the most common bird species in our data as well as in (military) aviation on a European scale (Dekker et al. 2003). Buzzards are considered vulnerable to collisions with power lines and wind turbines as well (Rose and Baillie 1989; Bose et al. 2020). However, a GPS‐tracking study on common buzzards on military airbases in the Netherlands (Van Gasteren et al. 2014) showed that birds nesting close to the runway never collided with fighter jets. This may be due to small territories that did not overlap with the runway or due to familiarity with the local situation in combination with a cognitive ability to deal with the risks. In contrast to these territorial birds, nonbreeding birds held large territories, often including the entire airbase, and two out of six of these nonbreeding common buzzards collided with aircraft within 4 months after tagging (Van Gasteren et al. 2014). For several species that were common at the airfields, no bird strikes were registered. For these species, we assumed a bird strike ratio lower than one bird strike during the total amount of aircraft movements (Appendix 1; ratio set at 0.0035). For three species, this assumption resulted in rather low SSI values, whereas strike impacts will be high (Dark‐bellied Brent Goose (*Branta bernicla*), Greylag Goose (*Anser anser*), and Mute Swan (*Cygnus olor*)). The reason for these low SSI values, despite their high body mass, is probably related to the full focus of bird control units on corrective measures when these “dangerous” species are found on airbases. For other species that were present at the airfields but for which no strike data occurred in our database, a power regression between bird strike sensitivity and body mass (*R* ^2^ 0.493, *n* 97, *p* < 0.001) was used to provide an estimate for SSI.

### 4.2 BSRI as an Indicator of Effectiveness of Bird Strike Management

We developed BSRI to predict hazard risk at our military airbases at a certain moment, based on abundance data of all bird species in the runway area. BSRI indicates the hazard risks at any time for airbases with one runway or with two crossed runways. This method is therefore applicable to all military airbases and smaller regional airfields. For large civil airfields, with multiple runways, we suggest to apply BSRI for each runway separately. Here, BSRI can be used as a flight safety performance indicator, for example, to switch runways.

Over the last 10 years, BSRI decreased at five out of six RNLAF airbases. This suggests that the bird strike management activities at RNLAF airbases are effective, that is, habitat management of low‐nutrient grasslands combined with corrective measures taken by bird control units. Habitat management was adjusted substantially in 1996, after which the grasslands changed over the years from intensively managed grasslands to herbaceous grasslands and on sandy soils to species‐rich *Nardus* grasslands (with *Festuca* and heather) (Schippers et al. 2023). This grassland management regimen has affected the bird species composition. This change in composition is reflected in, for instance, a reduction of flocking bird species. Although these species have low SSI values as expressed per individual, their flocking behavior and thus higher abundance resulted in high BSRI values and thus indicated high hazard risks. Typical examples of these flocking species are Northern Lapwings (*Vanellus vanellus*), Black‐headed Gulls (*Chroicocephalus ridibundus*), Common Gulls (*Larus canus*), and Starlings. Simultaneously, as a result of grassland management, numbers of small songbirds increased in the breeding season, such as non‐flocking Eurasian Skylarks (*Alauda arvensis*). These have both low SSI and low BSRI and therefore do not present a higher hazard risk. Unfortunately, in recent years, grassland management has also led to an increase in birds of prey, mainly common buzzard (higher SSI) and common kestrel (low SSI), that can increase BSRI when present in high numbers. Common kestrels have a low strike impact but are prone to bird strikes, and their presence on airfields may not so much increase hazard risk but can potentially negatively affect already declining population levels. Despite the increase in numbers of birds of prey on airbases, the overall BSRI has decreased, supporting earlier findings that birds of prey are less harmful to air operations than flocking species such as lapwings, gulls, or geese (Dekker 2010).

### 4.3 Seasonal Patterns in BSRI

Over the season, BSRI values reflect ecological and behavioral aspects of different bird species at the airbases. Various examples can explain these seasonal patterns in BSRI and show that BSRI and SSI together accurately reflect the hazard risk at our airbases: (1) low BSRI values as a result of breeding season (April–May) and the absence of migratory birds. Although Skylarks nest in high densities at RNLAF airbases (∼75–100 territories per airbase (Van Heusden et al. 2023), the low SSI resulted in low BSRI values, reflecting the lack of damage caused to aircraft by this species; (2) black‐headed Gulls and Mediterranean Gulls (*Ichthyaetus melanocephalus*) temporarily foraging in high numbers on emerging Garden Chafers (*Phyllopertha horticola*), resulted in an increased BSRI in June for Woensdrecht; (3) elevated numbers of foraging Stock Doves (*Columba oenas*) from mid‐June to mid‐July resulted in an increased BSRI on Woensdrecht, Eindhoven, and Volkel; and (4) the post‐fledging and pre‐migratory period of common buzzard and common kestrel from mid‐July to mid‐September on Eindhoven, Leeuwarden, and Volkel led to an increased BSRI and resulted in the need for bird control units to regularly remove birds of prey, by catch‐and‐release‐elsewhere approaches or as a last resort cull individual birds. The seasonal BSRI values for each airbase allowed the bird control units to visualize and anticipate on the riskiest periods in the year. BSRI can thus be helpful to optimize management measures, such as the timing of mowing and infrastructural works.

### 4.4 BSRI as a Leading Indicator

BSRI offers a strong and straightforward leading indicator for bird risk management. Our bird‐count‐based method does not include relative behavioral or ecological variables. These variables are instead incorporated in the calculation by using individual species abundance data and body mass. This makes BSRI accurate and less sensitive to uncertainty, as knowledge on behavior and ecology is difficult to quantify. High risk situations, resulting from, for example, seasonality or behavior, are thus reflected in BSRI via higher bird counts. This bird‐count‐based method in local bird strike management can be used for a wide range of possible applications that are useful in daily practice and operation. When bird numbers are recorded, each bird inspection of the runway environment results in a BSRI value and thus in a local bird status (normal, alert, or critical) based on the locally adopted threshold values. This instantaneous BSRI value can support bird control personnel in their day‐to‐day bird control actions. Practical applications would be changing the local procedures for air operations (e.g., switching runways, no local training of landing and takeoffs, closing runways) as well as for bird control units (e.g., starting dispersal actions, more frequent inspections, and extra personnel), dependent on the local BSRI values and thresholds. On the longer term, monthly values of BSRI resulting in alert or critical bird statuses due to increased abundance of specific species can give insight in risk developments on the airfield. This facilitates the implementation of species‐specific bird control actions. For instance, in the RNLAF, BSRI‐threshold critical is used as a measure to start catching birds of prey and release them distances away (>25 km). Over time, trends in yearly and seasonal patterns provide valuable information on effectiveness of preventive measures in terms of grassland management. At the same time, these trends in BSRI provide us with insights in historical developments as well as allow us to develop future strategies.

### 4.5 Limitations

#### 4.5.1 Data Availability

Before the risk assessment method proposed in this article can be implemented at an airfield, an adequate counting and reporting system of both (daily) bird abundance and bird strikes on the airfield is essential. This reporting system should at least require information on species in both count and strike data. If not available, applying the risk assessment method is not possible. Nevertheless, the implementation of an adequate reporting system is stated as a wildlife management requirement in ICAO Doc 9137 (2020), and thus most airfields worldwide will fulfill these requirements.

#### 4.5.2 Aircraft Type

The impact of bird species varies across aircraft types (e.g., Pfeiffer et al. 2018), which would suggest that SSI should also be calculated per aircraft type. However, Pfeiffer et al. also showed that lumping all military aircraft together still resulted in a strong relationship between hazard score and body mass of different bird species. In our study, we aim to provide a simple metric based on bird count and species composition (BSRI), which is easily applicable for bird control units and/or air traffic control and must be straightforward and easy to interpret (thresholds for alert and critical showing an increased bird strike risk).

#### 4.5.3 Threshold Values

The threshold BSRI values at which an airfield will change their safety status to alert or critical will be different for every airfield. We cannot determine for other airfields where these thresholds should lie. However, for RNLAF airbases, we used 3.1 (80%) and 3.7 (95%) as threshold values. These values could be determined on the basis of (1) an extensive dataset on both strikes and bird species involved, and (2) a relatively safe situation with low bird strike probability and severity on the RNLAF airfields, where preventive measures and bird control units have been standard operating procedure for several decades. At other airfields, these thresholds values will need to be defined depending on the local safety situation of the airfield. By lack of locally defined threshold values, the RNLAF values can be used (3.1 and 3.7 resp. for alert and critical). These values can be adjusted at airfields by bird control units, depending on the level of risk accepted locally.

## 5 Conclusion

Our approach to bird strike management is based on the actual abundance of birds on an airfield and serves as a framework to assess and reduce hazard risks from bird strikes. By introducing two straightforward and quantifiable indices, SSI and BSRI, risk assessment can become easier for airfields. With our Species Strike Impact (SSI), high‐ and low‐risk bird species are defined. This allows bird control units to take bird control measures for bird species that have high strike risks and high strike impacts (high SSI values). In addition, our BSRI provides a quantified insight in current and actual bird strike risk for all bird species present on the airfield. Our method thus provides a widely applicable leading indicator in flight safety management that can be used to assess and mitigate bird strike risk.

## APPENDIX 1

### 1.1

 [Table risa70101-tbl-0005]

**TABLE A1 risa70101-tbl-0005:** Overview of bird strike ratio, bird density, bird strike sensitivity, body mass, and Species Strike Impact for 97 bird species that were commonly observed and struck.

Species name	Bird strike ratio (#/10,000 movements)	Bird density (#/10 ha)	Bird strike sensitivity	body mass (kg)	Species Strike Impact (SSI)
Barn Owl—*Tyto alba*	0.007	0.00001	1219.0	0.231	1.638
Blackbird—*Turdus merula*	0.012	0.00230	5.3	0.089	0.163
Black‐headed Gull—*Chroicocephalus ridibundus*	0.230	0.14008	1.6	0.261	0.254
Black‐tailed Godwit—*Limosa limosa*	0.035	0.01964	1.8	0.315	0.321
Brambling—*Fringilla montifringilla*	0.005	0.00024	20.8	0.025	0.075
Buzzard—*Buteo buteo*	0.454	0.08465	5.4	0.860	1.591
Carrion Crow—*Corvus corone*	0.071	0.51776	0.1	0.520	0.067
Chaffinch—*Fringilla coelebs*	0.009	0.00232	3.9	0.022	0.035
Common Gull—*Larus canus*	0.060	0.19806	0.3	0.404	0.107
Common Tern—*Sterna hirundo*	0.003	0.00144	1.8	0.112	0.113
Cormorant—*Phalacrocorax carbo*	0.004	0.01010	0.4	2.254	0.807
Curlew—*Numenius arquata*	0.028	0.04878	0.6	0.725	0.333
Egyptian Goose—*Alopochen aegyptiaca*	0.004	0.00413	1.1	1.975	1.420
Feral Pigeon—*Columba livia domestica*	0.091	0.00813	11.2	0.370	0.927
Fieldfare—*Turdus pilaris*	0.002	0.03572	0.1	0.105	0.005
Garganey—*Spatula querquedula*	0.006	0.00006	104.9	0.326	1.520
Golden Plover—*Pluvialis apricaria*	0.018	0.00387	4.6	0.215	0.372
Goldfinch—*Carduelis carduelis*	0.006	0.00599	1.0	0.016	0.011
Great Black‐backed Gull—*Larus marinus*	0.005	0.00009	56.6	1.600	6.483
Great Spotted Woodpecker—*Dendrocopos major*	0.004	0.00071	5.2	0.074	0.134
Green Woodpecker—*Picus viridis*	0.002	0.00138	1.3	0.176	0.148
Greenfinch—*Chloris chloris*	0.010	0.00137	7.4	0.028	0.060
Grey Heron—*Ardea cinerea*	0.035	0.03072	1.2	1.433	1.098
Grey Plover—*Pluvialis squatarola*	0.002	0.00007	25.3	0.122	0.397
Herring Gull—*Larus argentatus*	0.053	0.01917	2.8	1.150	1.530
Hobby—*Falco subbuteo*	0.028	0.00026	107.6	0.215	1.006
House Martin—*Delichon urbicum*	0.007	0.00349	1.9	0.018	0.019
Jackdaw—*Coloeus monedula*	0.028	0.42518	0.1	0.229	0.015
Jay—*Cyanocitta cristata*	0.005	0.01743	0.3	0.168	0.046
Kestrel—*Falco tinnunculus*	1.188	0.05819	20.4	0.213	0.651
Lapwing—*Vanellus vanellus*	0.159	0.27522	0.6	0.219	0.100
Lesser Black‐backed Gull—*Larus fuscus*	0.018	0.01925	0.9	0.817	0.534
Linnet—*Linaria cannabina*	0.022	0.02677	0.8	0.020	0.012
Little Grebe—*Tachybaptus ruficollis*	0.002	0.01515	0.1	0.201	0.023
Little Owl—*Athene noctua*	0.002	0.00001	315.0	0.192	1.102
Little Ringed Plover—*Charadrius dubius*	0.007	0.00014	52.6	0.039	0.153
Long‐eared Owl—*Asio otus*	0.009	0.00012	76.4	0.276	1.200
Magpie—*Pica pica*	0.011	0.07945	0.1	0.219	0.027
Mallard—*Anas platyrhynchos*	0.020	0.11710	0.2	1.119	0.179
Marsh Harrier—*Circus aeruginosus*	0.005	0.00130	3.8	0.639	1.007
Meadow Pipit—*Anthus pratensis*	0.018	0.04677	0.4	0.018	0.006
Mediterranean Gull—*Ichthyaetus melanocephalus*	0.012	0.00247	4.7	0.273	0.473
Merlin—*Falco columbarius*	0.007	0.00004	187.8	0.206	1.080
Mistle Thrush—*Turdus viscivorus*	0.009	0.02730	0.3	0.109	0.032
Moorhen—*Gallinula chloropus*	0.002	0.02568	0.1	0.285	0.020
Oystercatcher—*Haematopus ostralegus*	0.068	0.10400	0.7	0.541	0.272
Partridge—*Perdix perdix*	0.018	0.01845	1.0	0.386	0.266
Peregrine—*Falco peregrinus*	0.002	0.00013	13.9	0.890	2.401
Pintail—*Anas acuta*	0.003	0.00013	19.3	0.838	2.523
Quail—*Coturnix coturnix*	0.002	0.00034	5.4	0.099	0.184
Redwing—*Turdus iliacus*	0.011	0.00695	1.6	0.062	0.059
Reed Bunting—*Emberiza schoeniclus*	0.005	0.00126	4.3	0.020	0.033
Ringed Plover—*Charadrius hiaticula*	0.007	0.00001	917.8	0.062	0.423
Rook—*Corvus frugilegus*	0.015	0.17783	0.1	0.498	0.040
Rough‐legged Buzzard—*Buteo lagopus*	0.005	0.00025	20.1	1.102	3.360
Sand Martin—*Riparia riparia*	0.014	0.00351	4.1	0.014	0.022
Shelduck—*Tadorna tadorna*	0.005	0.00895	0.6	1.063	0.470
Siskin—*Spinus spinus*	0.004	0.00022	16.3	0.013	0.037
Skylark—*Alauda arvensis*	0.229	0.19148	1.2	0.041	0.032
Snipe—*Gallinago gallinago*	0.002	0.00228	0.8	0.120	0.070
Song Thrush—*Turdus philomelos*	0.002	0.00061	3.0	0.068	0.094
Sparrow Hawk—*Accipiter nisus*	0.007	0.00097	7.5	0.208	0.445
Starling—*Sturnus vulgaris*	0.128	1.11219	0.1	0.079	0.009
Stock Dove—*Columba oenas*	0.304	0.11982	2.5	0.291	0.367
Swallow—*Hirundo rustica*	0.307	0.10742	2.9	0.019	0.026
Swift—*Apus apus*	0.385	0.01502	25.7	0.043	0.141
Tree Sparrow—*Passer montanus*	0.005	0.00040	13.6	0.024	0.063
Turtle Dove—*Streptopelia turtur*	0.002	0.00034	5.4	0.138	0.257
Wheatear—*Oenanthe oenanthe*	0.016	0.00722	2.2	0.025	0.029
Whimbrel—*Numenius phaeopus*	0.005	0.00489	1.0	0.463	0.325
White Stork—*Ciconia ciconia*	0.002	0.00014	13.5	3.557	9.505
White Wagtail—*Motacilla alba*	0.002	0.02067	0.1	0.021	0.002
Woodcock—*Scolopax rusticola*	0.006	0.00005	113.3	0.315	1.493
Woodlark—*Lullula arborea*	0.012	0.00132	8.8	0.029	0.066
Woodpigeon—*Columba palumbus*	0.148	0.28939	0.5	0.520	0.215
**Bird species with no bird strikes and bird density ≥0.001**					
Black‐necked Grebe—*Podiceps nigricollis*	<0.0035	0.01573	0.2	0.321	0.065
Canada Goose—*Branta canadensis*	<0.0035	0.00141	2.5	4.635	5.791
Common Pheasant—*Phasianus colchicus*	<0.0035	0.01245	0.3	1.278	0.317
Common Pochard—*Aythya ferina*	<0.0035	0.00289	1.2	0.976	0.774
Common Redshank—*Tringa totanus*	<0.0035	0.00324	1.1	0.129	0.095
Dark‐bellied Brent Goose—*Branta bernicla*	<0.0035	0.09650	0.1	1.411	0.050
Eurasian Goshawk—*Accipiter gentilis*	<0.0035	0.00134	2.6	1.140	1.464
Eurasian Teal—*Anas crecca*	<0.0035	0.00780	0.4	0.325	0.120
Eurasian Wigeon—*Mareca penelope*	<0.0035	0.00371	0.9	0.700	0.465
Eurasian Coot—*Fulica atra*	<0.0035	0.24325	0.1	0.899	0.013
European Stonechat—*Saxicola rubicola*	<0.0035	0.00509	0.7	0.017	0.009
Gadwall—*Mareca strepera*	<0.0035	0.03232	0.1	0.810	0.083
Great Crested Grebe—*Podiceps cristatus*	<0.0035	0.00197	1.8	1.061	1.083
Green Sandpiper—*Tringa ochropus*	<0.0035	0.00152	2.3	0.069	0.081
Greylag Goose—*Anser anser*	<0.0035	0.04170	0.1	3.225	0.260
Hen Harrier—*Circus cyaneus*	<0.0035	0.00121	2.9	0.411	0.559
Mute Swan—*Cygnus olor*	<0.0035	0.01905	0.2	9.675	1.632
Northern Shoveler—*Spatula clypeata*	<0.0035	0.00380	0.9	0.600	0.392
Sedge Warbler—*Acrocephalus schoenobaenus*	<0.0035	0.00146	2.4	0.013	0.016
Tree Pipit—*Anthus trivialis*	<0.0035	0.00193	3.4	0.024	0.035
Tufted Duck—*Aythya fuligula*	<0.0035	0.05895	0.1	0.679	0.039
Western Yellow wagtail—*Motacilla flava*	<0.0035	0.01015	0.3	0.017	0.005

## APPENDIX 2

### 2.1

 [Table risa70101-tbl-0006]

**TABLE A2 risa70101-tbl-0006:** Confusion matrix based on inspection counts with local bird status as reference and estimated status dependent on Bird Strike Risk Index (BSRI) as predicted.

		Reference (local bird status)			
		Normal	Alert	Critical	Total
**Predicted (BSRI)**	**Normal**	147,742	15,925	1657	165,324
	**Alert**	11,733	4020	1574	17,327
	**Critical**	798	602	1079	2479
	**Total**	160,273	20,547	4310	**18,5130**
	**Sensitivity**	0.922	0.196	0.250	
	**Accuracy**	82.6%			

*Note*: Sensitivity and accuracy statistics provided in the table.
